# Proteomic dataset of the organohalide-respiring bacterium Dehalococcoides mccartyi strain CBDB1 grown on hexachlorobenzene as electron acceptor

**DOI:** 10.1016/j.dib.2016.02.037

**Published:** 2016-02-22

**Authors:** Christian L. Schiffmann, Wolfgang Otto, Rasmus Hansen, Per Halkjær Nielsen, Lorenz Adrian, Jana Seifert, Martin von Bergen, Nico Jehmlich

**Affiliations:** a Department of Molecular Systems Biology, Helmholtz Centre for Environmental Research–UFZ, Permoserstr. 15, 04318 Leipzig, Germany; b Aalborg University, Department of Biotechnology, Chemistry and Environmental Engineering, Sohngårdsholmsvej 49, 9000 Aalborg, Denmark; c Department Isotope Biogeochemistry, Helmholtz Centre for Environmental Research–UFZ, Permoserstr. 15, 04318 Leipzig, Germany; d Institute of Animal Science, Hohenheim University, 70593 Stuttgart, Germany

**Keywords:** Proteomic reference profile, Dehalococcoides mccartyi strain, CBDB1, Shotgun proteomics, Spectral library

## Abstract

The proteome of the anaerobic organohalide-respiring bacterium *Dehalococcoides mccartyi* strain CBDB1 was analyzed by nano liquid chromatography coupled to mass spectrometry (LC-MS/MS). Two different preparation methods, (i) in-solution and (ii) in-gel proteolytic digestion were assessed to elucidate the core and the functional proteome of bacterial cultures grown in synthetic anaerobic medium with hexachlorobenzene as sole electron acceptor. A detailed analysis of the data presented is available (Schiffmann et al., 2014) [1].

**Specifications table**

**Table:** 

Subject area	Microbiology
More specific subject area	Microbiology of anaerobe organohalide respiring bacteria
Type of data	Mass spectrometry (MS) data
How data was acquired	Data was recorded using an nano-liquid chromatography coupled Q Exactive instrument
Data format	Raw LC-MS data (*.raw), protein identifications as Magellan storage files (*.msf) and spectral library (*.splib/*.zip)
Experimental factors	*Dehalococcoides mccartyi* strain CBDB1 was grown in synthetic anaerobic medium with 5 mM acetate as carbon source, hydrogen as electron donor and hexachlorobenzene as sole electron acceptor.
Experimental features	Peptide extracts were prepared by in-solution or in-gel proteolytic digestion. Raw data was searched using the *D. mccartyi* strain CBDB1 database retrieved from UniProt/Swiss-Prot. A library for spectral searches was created.
Data source location	Leipzig, Germany
Data accessibility	Data is deposited at ProteomeXchange via the PRIDE partner repository with the dataset identifier PXD003081.

**Value of the data**

• The extensive proteome set of *Dehalococcoides mccartyi* strain CBDB1 can be used for comparative experiments with reductive dehalogenation**.**
• Spectral library can be applied as an additional tool for database searches to increase the confidence and reliability of peptide identifications.
• LC-MS data and protein identifications can be used as repository for new functional insights into the reductive dehalogenation process and to classify the proteins according to physiological functions and subcellular localization.

## 1 Data

*Dehalococcoides mccartyi* strain CBDB1 is an obligate organohalide respiring organism and a model organism for this mode of energy conservation. The mass spectrometric (MS) data deposited in the ProteomeXchange archive (PXD003081) represents the first comprehensive approach to characterize the proteome of *D. mccartyi* strain CBDB1 grown with hexachlorobenzene as sole electron acceptor [1]. Besides the raw LC–MS data, protein identification files and a spectral library are provided. Studies focused on certain aspect of the metabolism had previously shown the value of MS approaches for *D. mccartyi* strain CBDB1, which hardly yields enough biomass for classical biochemical methods [2], [3], [4]. The identification of 70% of the annotated 1458 protein-coding sequences makes this proteomic data a valuable reference, giving evidence for the transcription and translation of coding sequences previously only predicted by genome annotation. Using the peptide identification information of mass spectra, a spectral library was compiled. This library can be applied to improve peptide identification in results from data independent acquisitions or as database for the design of targeted MS methods.

## 2 Experimental design, materials and methods

### 2.1 Cultivation and sampling

*D. mccartyi* strain CBDB1 was grown in synthetic, Ti(III) citrate-reduced anaerobic medium containing 5 mM acetate as carbon source. Hexachlorobenzene crystals were provided as electron acceptor and hydrogen as electron donor with a nominal concentration of 7.5 mM as described [5]. A total volume of 280 ml cell culture was harvested using sterile filters (0.2 µm pore diameter, Millipore, Billerica, MA, USA). Cells were washed from the filter with 50 mM ammonium hydrogen carbonate. Aliquots representing 70 ml culture were stored at −20 °C.

### 2.2 Sample preparation

Samples were prepared by two different methods for mass spectrometry: Prefractionation of proteins by one dimensional sodium dodecyl sulfate polyacrylamide gel electrophoresis (SDS-PAGE) combined with in-gel digestion or in-solution digest. For in-solution digest washed and concentrated *D. mccartyi* strain CBDB1 cells were lysed by three cycles of freeze (liquid nitrogen) and thaw (1 min shaking at 40 °C). Resulting protein lysates were reduced in 50 mM dithiothreitol (Invitrogen, Carlsbad, CA, USA) for 1 h at 30 °C followed alkylation in 130 mM iodacetamide (Sigma-Aldrich, St. Louis, Mo, USA) for 1 h at 30 °C. Protein digestion by sequencing-grade trypsin (Promega, Madison, WI, USA) was carried out overnight at 37 °C. The reaction was stopped by adding formic acid to a final concentration of 1%. Peptides were extracted and desalted using ZipTip-μC18 tips (Merck Millipore, Darmstadt, Germany). After evaporation of solvents, samples were stored at −20 °C until further processing.

For SDS-PAGE, aliquots of washed and concentrated *D. mccartyi* strain CBDB1 cells were resuspended in denaturizing preparation buffer for 10 min at 90 °C in duplicates. Gel run was conducted for 30 min at 20 mA following 15 min at 40 mA. The protein containing lanes were cut into ten slices, for each slice in-gel digest was performed separately. Therefore after reduction and alkylation proteins were digested overnight at 37 °C using sequencing-grade trypsin. Peptides were extracted and desalted using μC18 ZipTip tips. After evaporation of solvents, peptide extracts were stored at −20 °C until further analysis.

### 2.3 Mass spectrometric analysis

For LC-MS/MS analysis samples were resuspended in 0.1% formic acid 2% acetonitrile (ACN). Liquid chromatography was carried out using an Ultimate 3000 RSLC (Thermo Scientific Dionex) system. A sample volume of 7 µl resuspended peptides was trapped onto a column with 75 μm inner diameter packed with 3 μm C18 particles (Acclaim PepMap100, Thermo Scientific). Reverse phase separation was carried out with a constant flow of 300 nL/min over a 50 cm analytical column packed with 2 μm C18 particles (Acclaim PepMap RSLC, Thermo Scientific). Both columns were tempered to 40 °C at all times. The applied linear gradient of buffer A (0.1% formic acid, 0.005% heptafluorobutyric acid) to B (90% acetonitrile, 0.1% formic acid, 0.005% heptafluorobutyric acid) was 10–45% within 60 min for each of the SDS-PAGE samples and to the same concentration within 180 min for the in-solution digested samples. Eluting peptides were analyzed in data dependent mode on a Q Exactive (Thermo Fisher Scientific; Bremen, Germany). Survey scans were recorded in the Orbitrap analyzer with a resolution of 70,000 using an ion target value of 3×10^6^ and maximum ion injection time of 150 ms. Based on intensity up to ten ions with charge ≥2 were picked for further analysis. Isolated with a window of 3 m/z, precursor ions were subjected to higher energy collisional dissociation (HCD) with normalized collision energy of 30%. Ion target value was defined as 2×10^5^ with a maximum fill time of 150 ms. In order to prevent repeated analysis of the same peptide ions, dynamic exclusion was applied for duration of 30 s.

### 2.4 Data analysis

Data resulting from LC-MS/MS experiments were analyzed using the Proteome Discoverer v1.4.0.288 (Thermo Scientific) with the algorithms SEQUEST and MS Amanda for peptide identification. Spectra were matched to the *D. mccartyi* strain CBDB1 database containing 1454 unique sequences (UniProt, 11/2011). The database search was carried out with the following settings: cleavage enzyme trypsin, up to two missed cleavages allowed, precursor mass tolerance and fragment mass tolerance were set to 5 ppm and 0.05 Da, respectively. Oxidation of methionine was selected as dynamic and carbamidomethylation on cysteine residues as fixed modification. The false discovery rate (FDR) of identified peptide sequences was kept to <1% (using Percolator). To build the spectral library, LC-MS/MS data was processed using the ISB/SPC Trans Proteomic Pipeline (TPP v4.6 OCCUPY rev 2, Build 201302140902 (MinGW)). Accurate mass binning, decoy distribution and filtering of the results (PeptideProphet probability >0.9, quality filters according to [6]) were applied.

## Conflict of interest

None.
