# Supplementary material for: Proteomic dataset of the organohalide-respiring bacterium Dehalococcoides mccartyi strain CBDB1 grown on hexachlorobenzene as electron acceptor
Source: Data Brief. 2016 Feb 22;7:253–6. doi: 10.1016/j.dib.2016.02.037 (PMC4773567; doi:10.1016/j.dib.2016.02.037)
Supplement: Supplementary file 1 — Supplementary material [file mmc1.docx]

**Conflict of Interest**

All authors declare to have no conflict of interest.
